# An Engineered Infected Epidermis Model for In Vitro Study of the Skin’s Pro-Inflammatory Response

**DOI:** 10.3390/mi11020227

**Published:** 2020-02-23

**Authors:** Maryam Jahanshahi, David Hamdi, Brent Godau, Ehsan Samiei, Carla Liria Sanchez-Lafuente, Katie J. Neale, Zhina Hadisi, Seyed Mohammad Hossein Dabiri, Erik Pagan, Brian R. Christie, Mohsen Akbari

**Affiliations:** 1 Laboratory for Innovations in MicroEngineering (LiME), Department of Mechanical Engineering, University of Victoria, Victoria, BC V8P 5C2, Canada; jahanshahi1993@gmail.com (M.J.); david1hamdi@gmail.com (D.H.); brentgodau@gmail.com (B.G.); e.samiei@gmail.com (E.S.); zhinahadisi@gmail.com (Z.H.); mohammadhossein.dabiri@gmail.com (S.M.H.D.); erikpm@uvic.ca (E.P.); 2 Division of Medical Sciences, University of Victoria, Victoria, BC V8P 5C2, Canada; carlaliria@uvic.ca (C.L.S.-L.); katiejneale@gmail.com (K.J.N.); brain64@uvic.ca (B.R.C.)

**Keywords:** epidermis, 3D bioprinting, wound modeling, infection, pro-inflammatory response

## Abstract

Wound infection is a major clinical challenge that can significantly delay the healing process, can create pain, and requires prolonged hospital stays. Pre-clinical research to evaluate new drugs normally involves animals. However, ethical concerns, cost, and the challenges associated with interspecies variation remain major obstacles. Tissue engineering enables the development of in vitro human skin models for drug testing. However, existing engineered skin models are representative of healthy human skin and its normal functions. This paper presents a functional infected epidermis model that consists of a multilayer epidermis structure formed at an air-liquid interface on a hydrogel matrix and a three-dimensionally (3D) printed vascular-like network. The function of the engineered epidermis is evaluated by the expression of the terminal differentiation marker, filaggrin, and the barrier function of the epidermis model using the electrical resistance and permeability across the epidermal layer. The results showed that the multilayer structure enhances the electrical resistance by 40% and decreased the drug permeation by 16.9% in the epidermis model compared to the monolayer cell culture on gelatin. We infect the model with *Escherichia coli* to study the inflammatory response of keratinocytes by measuring the expression level of pro-inflammatory cytokines (interleukin 1 beta and tumor necrosis factor alpha). After 24 h of exposure to *Escherichia coli*, the level of IL-1β and TNF-α in control samples were 125 ± 78 and 920 ± 187 pg/mL respectively, while in infected samples, they were 1429 ± 101 and 2155.5 ± 279 pg/mL respectively. However, in ciprofloxacin-treated samples the levels of IL-1β and TNF-α without significant difference with respect to the control reached to 246 ± 87 and 1141.5 ± 97 pg/mL respectively. The robust fabrication procedure and functionality of this model suggest that the model has great potential for modeling wound infections and drug testing.

## 1. Introduction

Skin is the largest human organ, and serves as a physiological and immunologic barrier to protect the body from ultraviolet (UV) light, pathogens, environmental pollutants, and micro-organisms. Injuries, infected wounds, and scars can disturb skin’s functions and result in more severe physiological and psychological problems [1,2,3,4]. Currently, wound infection remains a primary concern worldwide, and a considerable share of the annual health budget is dedicated to wound treatment. In the United States alone, over 6.5 million patients are suffering from skin wounds, and the treatment of skin-related diseases costs over $25 billion US. Increasing the number of infected wounds and challenges associated with antibiotic resistance necessitate the development of more effective drugs and drug delivery systems [5].

Current drug development research relies mainly on animals [6], ex vivo [7,8,9,10,11], and in vitro cell culture models [12]. Animal models are associated with several challenges, including inter-species discrepancies, ethical and regulatory issues, and high costs [13,14,15]. According to Humane Society International, 9 out of 10 drugs with safe and effective results in animal studies fail in clinical trials [3,8]. Ex vivo human skin obtained from surgical procedures has been used for years as a model to study skin physiology and drug testing [4]. However, difficulty in finding donors coupled with the short shelf life of excised tissues hinder their use for high throughput studies [4,7]. These issues have led to the widespread use of in vitro two-dimensional (2D) models, in which a monolayer of skin cells is cultured in well plates and used to study the cellular response to drugs. The results from in vitro studies with 2D models can serve as the basis for decisions whether or not to proceed with the drug development process. However, these monolayer models are not reliable for predicting cell-specific responses to new compounds due to the lack of native in vivo morphology, cell-cell communication, and cell-matrix interactions [4,16].

Recent advances in tissue engineering and advanced manufacturing techniques have enabled researchers to create biomimetic tissues in a high-throughput fashion [17,18]. These models start from multilayered sheets of keratinocytes [4] and evolve to reconstructed, full-thickness human skin models using cell inserts [19,20], microfluidics [3,14], and 3D printing [21,22]. In addition to the epidermal layer, full-thickness skin models consist of dermal and hypodermal equivalent sections. More complex structures can include more details, such as vascularization [15,21] or neural cells [4]. Using cell inserts is the simplest method for creating multilayered epidermis and dermis. However, more functional structures are obtained with materials that have mechanical properties that are closer to those of the natural skin and provide the cells with more meaningful cell-matrix interactions [12,23]. Examples of materials used for modeling skin are collagen [15,19,22,24,25], silk [26], fibrinogen [15], and decellularized extracellular matrix [21] Although using 3D bioprinting and microfluidic technologies increases the throughput and reduces the use of expensive reagents and cells in the drug screening process, their applications have been limited to evaluating the effects of new compounds in healthy skin [24,27]. Therefore, there is still a need to develop an infected wound model for studying the healing mechanism, pro-inflammatory responses, and the efficacy of new therapeutic agents.

**Figure 1:**
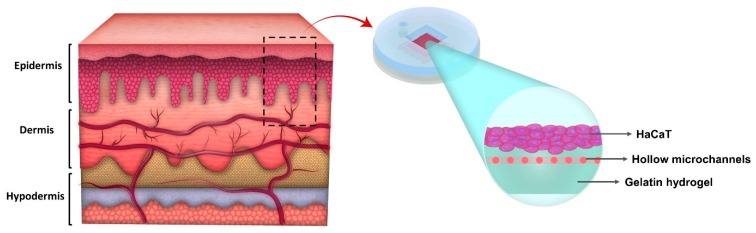
Simplified skin model including the epidermal layer as the first and main barrier of skin.

In this work, we developed a simplified functional skin model, resembling a skin barrier function to use in studying wound infection, pro-inflammatory response, and drug testing. We started with developing the epidermal layer, because it is the main physical barrier against drug delivery and plays an important role in activating the innate immune system when in contact with pathogens (Figure 1). We used gelatin as the matrix on which the cells reside because it is biocompatible, has low cost, and contains the major components of the skin’s extracellular matrix (ECM) including arginine-glycine-aspartic acid (RGD) sequences that promote cell attachment [28,29], and the target sequences of matrix metalloproteinase (MMP) that are suitable for cell remodeling [30]. We created hollow microchannels resembling microvessels by printing a thermos-responsive hydrogel as a sacrificial material within gelatin. Keratinocytes, the main cell type of the epidermis, were cultured for 6 weeks, and the terminally differentiated cells formed a multilayer structure. We infected the epidermis model using *Escherichia coli (E. coli*). *E. coli* is one of the most abundant gram-negative bacteria living on human skin [31,32]. It can cause infection in injured and burned tissues [33]. It is also widely used in studying infected wounds and the evaluation of antimicrobial agents [34,35,36,37]. In this work, *E. coli* was used to study the pro-inflammatory response of keratinocytes to infection and drug testing.

## 2. Results and Discussion

### 2.1. Model Development

**Figure 2:**
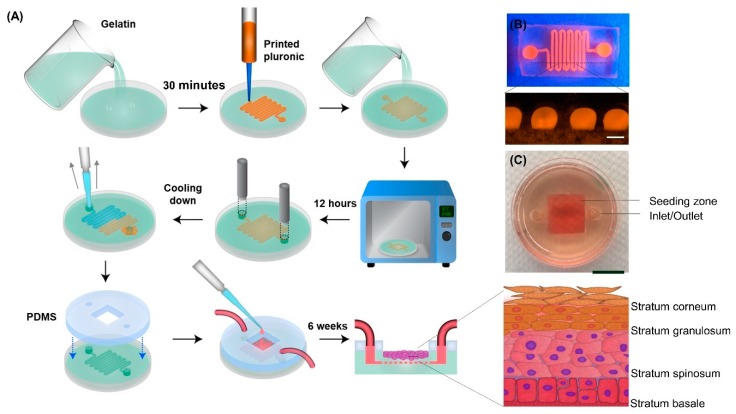
Model development and fabrication process. (**A**) Gelatin/ transglutaminase (TG) solution was poured into a 35 mm petri dish. After partial gelation, 38% pluronic F127 solution was printed on the gelatin layer. Subsequently, another layer of TG/gelatin solution was poured on the printed pattern and the hydrogel was incubated at 37 °C for 12 h. After punching an inlet and outlet, the hydrogel was cooled down and the liquid pluronic was removed from channel. Finally, a sterilized polydimethylsiloxane (PDMS) mold was mounted on the gelatin. After 6 weeks culture at the air-liquid-interface, immortalized human keratinocytes (HaCaT) cells formed a multilayer structure. (**B**) Cross-sectional view of the channel embedded in gelatin hydrogel. Scale bar is 500 µm. (**C**) Overview of the final model for submerged culture. Scale bar is 1 cm.

We have developed a novel gelatin-based epidermis model with artificial vasculatures embedded into the gelatin substrate (Figure 2). The vasculature network is pivotal to maintaining cells’ functions by providing essential nutrients and oxygen for cells and removing metabolites [38]. Moreover, the vasculature network in a skin model facilitates the in vitro investigation of drug permeation through the skin model and the drug’s potential to reach the circulatory system [39]. Among existing methods of creating the vasculature network, the 3D printing technique offers promising methods to generate the vasculature network in a highly controlled and automated manner for high throughput studies [40]. To create the vasculature network, a gelatin and transglutaminase (gelatin/TG) solution was loaded into a 35 mm petri dish. After partial gelation (30 min at room temperature), 38% Pluronic F127 solution was printed on the gelatin layer in a predesigned pattern using an extrusion-based 3D printer. The extrusion-based 3D printer was used due to its ability of depositing uninterrupted cylindrical fibers by applying a continuous air force [41,42]. Immediately after printing Pluronic, another layer of gelatin/TG solution was poured on the printed pattern and the hydrogel was incubated at 37 °C for 12 h. Afterward, an inlet and outlet were punched into the hydrogel using a 5 mm biopsy punch. Subsequently, the hydrogel was cooled down and the liquid Pluronic was removed from the outlet with a pipette. The resulting hollow channel serves as artificial vasculature. Finally, a sterilized polydimethylsiloxane (PDMS) mold was mounted on the gelatin to confine the seeding surface to a 1 cm × 1 cm area above the printed channels (so called seeding zone).

Gelatin was selected for the epidermis tissue model due to its high biocompatibility and tunable mechanical properties. Gelatin is a denatured form of collagen, produced by controlled hydrolysis of fibrous collagen. Gelatin mainly consists of triple amino acids of glycine, proline, and hydroxyproline. Gelatin is used as an additive in different biomaterials to enhance cell–scaffold interactions through Arginine-Glycine-Aspartate (RGD) motifs recognized by integrin receptors on cell membranes. Moreover, it is more cost effective and less antigenic than collagen [23,43]. In this study, gelatin was crosslinked enzymatically using transglutaminase. Transglutaminase (TG) is a natural enzyme derived from streptomycetes and can be activated over a wide range of temperatures and pHs [44,45]. It has been reported that TG is non-toxic and causes no side-effects on several cell types [44]. Using TG not only enables tuning gelatin’s mechanical properties and gelation time, but also eliminates the use of photo-initiators and ultraviolet (UV) exposure, which decrease cell viability [44].

Pluronic F127 is a thermo-responsive hydrogel that undergoes a sol-gel transition at biologically-relevant temperatures (10–40 °C). Pluronic is also highly printable hydrogel due to the nature of micellar-packing gelation, which allows it to be extruded easily and maintain its shape [46,47,48]. Moreover, the broad range of its sol-gel transition temperature allows its viscosity to be stable at both room and human body temperature. Due to such properties, Pluronic F127 was selected as a sacrificial material to form the hollow channel with a diameter of 500–800 µm inside the gelatin.

Keratinocytes, as the main cell type of the epidermis, compose four distinct regions of epidermis with different stages of differentiation, including stratum corneum, stratum granulosum, stratum spinosum, and stratum basal (Figure 2) [49,50]. To form the multilayer structure of the epidermis, keratinocytes were seeded on the gelatin and cultured for 7 days to form a confluent monolayer. Then, the media was removed from the seeding zone and the differentiation media was delivered to the cells through the artificial vasculature to culture the cells at an air-liquid-interface.

### 2.2. Gelatin Hydrogel Characterization

#### 2.2.1. Mechanical Properties of Gelatin

**Figure 3:**
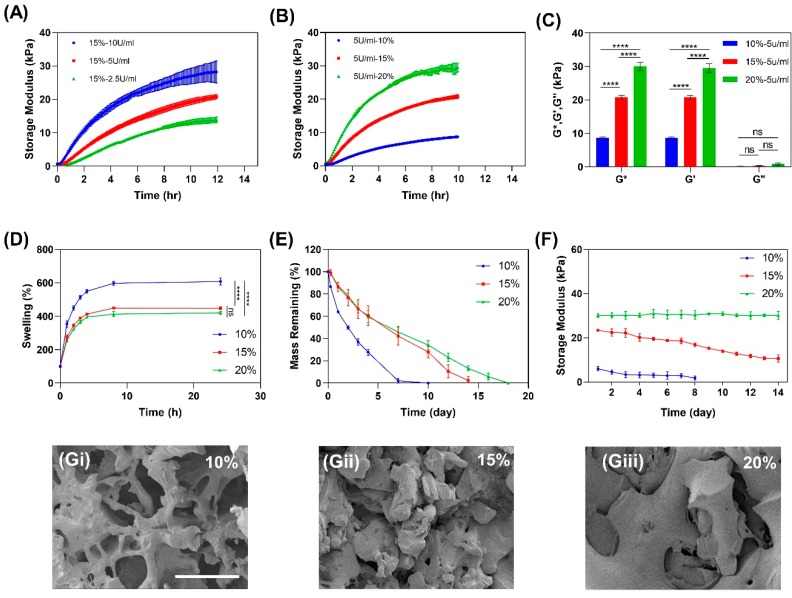
(**A**) Storage moduli of 15% gelatin crosslinking with 2, 5, and 10 U/mL of TG (n = 3). (**B**) Storage moduli of 10%, 15%, and 20% gelatin with 5 U/mL of TG (n = 3). (**C**) G*, Gʹ, and Gʺ of 10%, 15%, and 20% gelatin (n = 3). (**D**) Swelling ratio (n = 3). (**E**) Mass remaining percentage during degradation in 2 U/mL collagenase (n=3). (**F**) Storage moduli of 10%, 15%, and 20% gelatin during cell culture (n = 3). (**G**) Scanning electron microscopy (SEM) images of gelatin with 10% (**i**), 15% (**ii**), and 20% (**iii**) concentrations; scale bar is 200 µm. (Error bars indicate standard deviation. ns and **** indicate nonsignificant and *p* < 0.001, respectively.)

To study the effect of TG and gelatin concentration on mechanical properties of gelatin hydrogels, the storage modulus of gelatin was studied over crosslinking time. To crosslink gelatin enzymatically, TG was added to the gelatin solutions to obtain a biocompatible crosslinked hydrogel. TG catalyzes the formation of covalent bonds between the carbonyl and amino groups in gelatin. Crosslinking kinetics of 15% gelatin/TG were studied as a function of TG concentration by real-time measurement of the storage modulus (Gʹ) as an indicator of mechanical strength over a period of 12 h. Results show that the storage modulus continuously increased for all TG concentrations, while a higher level of storage modulus (28.2 ± 3.4 kPa) was observed as the concentration of TG increased to 10 U/mL, which was due to a greater extent of crosslinking (Figure 3A). However, 10 U/mL TG is associated with fast inhomogeneous gelation and difficulties in sample manipulation; therefore, 5 U/mL TG was selected for the remaining experiments. Figure 3B shows the effect of gelatin concentration with 5 U/mL TG on the storage modulus of the hydrogel. It was observed that increasing the gelatin concentration resulted in a higher storage modulus due to the higher densities of carbonyl and amino groups available for bonding to each other. This result is consistent with previous studies on the effects of gelatin and TG concentrations on gelatin hydrogel strength, showing the increase of gelatin mechanical strength by increasing the concentration of gelation or TG [28,44,51]. We found that 20% gelatin represented the closest storage modulus (24.0 ± 12.0 kPa) to human skin’s storage modulus, which is 40–60 kPa [52]. It should be noted that further increasing the gelatin concentration to achieve a higher storage modulus would be associated with several challenges with making a homogenous gelatin solution and casting it. Following the completion of gelation, the complex (G*) and loss (G”) moduli of the hydrogels were measured (Figure 3C) and it was observed that at all concentrations, the hydrogels showed dominant solid-like behavior (loss factor (Q^−1^) <1).

#### 2.2.2. Swelling Ratio

The swelling ratio of the hydrogels governed by the osmotic pressure is important, as it affects solute diffusion, surface properties, mechanical properties, and stability [53]. The swelling ratio is affected by the pore size of the polymer network, which is a function of gelatin concentration. To study the effect of gelatin concentration on swelling ratio, dried hydrogels with different gelatin concentrations were immersed in phosphate buffered saline (PBS) solution and weighed at each time point. As shown in Figure 3D, while the 10% gelatin hydrogel had a significantly higher swelling ratio than 15% and 20% hydrogels, it was observed that all hydrogels had high swelling ratios (around 600% for 10% and about 400% for 15% and 20% gelatin hydrogels)

#### 2.2.3. In Vitro Enzymatic Degradation

Degradation of tissue engineering scaffolds is required to make space for cell spreading and proliferation during culture [29]. However, the time and rate of degradation should be comparable with the rate of tissue formation [54]. To study the degradation rate, gelatin hydrogel disks with 16 mm diameter and 7 mm thickness were incubated with 2 U/mL collagenase solution at 37 °C. Figure 3E shows continuous weight loss in all samples due to the cleavage of peptide bonds within the gelatin structure. The results demonstrated that increase in the gelatin concentration reduced the degradation rate. The full dissolution of hydrogels enhanced from 7 days in 10% gelatin to 14 and 18 days for 15% and 20% gelatins, respectively. The higher crosslinking density is the main cause for the prolonged degradation of the 20% gelatin.

#### 2.2.4. Mechanical Stability of Gelatin Hydrogel in Culture

Scaffolds which are designed for cell culture and tissue formation need to exhibit proper mechanical properties and maintain them over the differentiation process to support cellular morphogenesis, proliferation, and differentiation [55]. In order to examine the stability of cell-seeded gelatin hydrogels and determine the impact of cell secreted materials on the integrity of hydrogels, immortalized human keratinocytes (HaCaT) with a density of 50,000 cell/cm^2^ were seeded on different concentrations of gelatin hydrogel. HaCaT cells were chosen because their in vitro differentiation and proliferation capabilities have been well established [28,56,57]. The storage modulus was then measured at different time points using ElastoSens™ Bio2 (Rheolution, Montreal, Quebec, Canada) (Figure 3F). This instrument works based on the mechanical vibration of a detachable sample holder containing a small amount of material, and the system deformation is measured by laser and converted into storage and loss moduli [58]. The results show that 10% gelatin lost its integrity after 7 days of incubation and detached from the sample holder due to degradation. For 15% gelatin, the hydrogel maintained its integrity for 14 days, although its storage modulus decreased by 50%. On the other hand, the storage modulus of 20% gelatin remained unchanged over 14 days of culture with respect to the storage modulus of non-cell-seeded gelatin (Figure 3B).

#### 2.2.5. Scanning Electron Microscopy of Gelatin Hydrogel

The porosity and interior microstructure of tissue engineering hydrogels affects the permeability, swelling ratio, and stiffness of the hydrogels in culture [59]. To visualize the microstructure of the hydrogels, scanning electron microscopy (SEM, Hitachi S4800, Tokyo, Japan) was used. Images show that increasing the concentration of gelatin results in a reduction of porosity (Figure 3G). The superior mechanical properties of 20% gelatin can also be attributed to the smaller pore size observed within the hydrogel (Figure 3Giii). These results suggest that mechanical properties and biodegradability of these hydrogels can be tuned by changing the concentration of gelatin and subsequently the degree of crosslinking.

#### 2.2.6. Cytocompatibility and Cell Attachment to Gelatin

**Figure 4:**
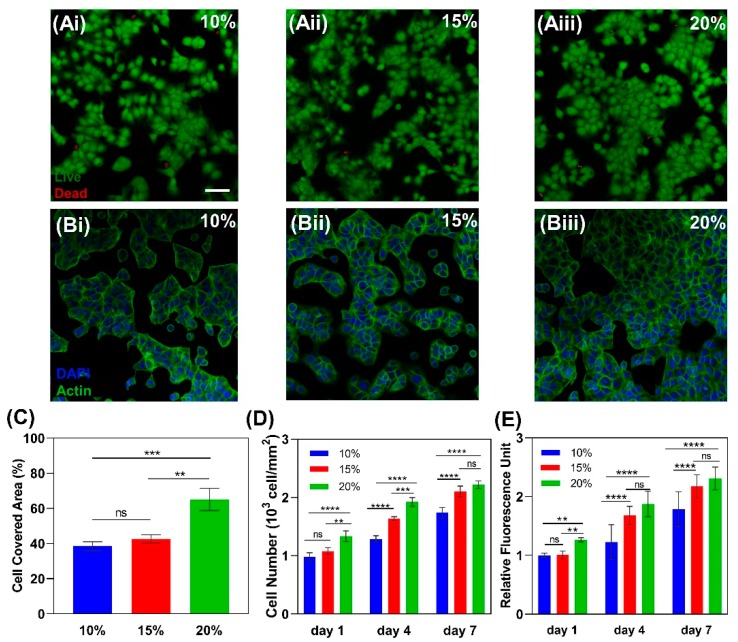
Viability, attachment, and proliferation of HaCaT cells cultured on gelatin hydrogels with different concentrations. (**A**) Representative live/dead fluorescence images of HaCaT cells on gelatin surfaces of 10% (**i**), 15% (**ii**), and 20% (**iii**) after 1 day of culture. Green fluorescent cells are alive and red fluorescent cells indicate dead ones. (**B**) Representative phalloidin/ 4′,6-diamidino-2-phenylindole (DAPI) fluorescence images of HaCaT cells on gelatin surfaces of 10% (**i**), 15% (**ii**), and 20% (**iii**) after 1 day of culture. Cell filaments are stained by phalloidin (green) and nuclei stained by DAPI (blue). (**C**) Quantification of cell covered area of different concentrations of gelatin 1 day after seeding using National Institute of Health (NIH) ImageJ software (n = 3). (**D**) Quantification of live cells using live/dead fluorescence images of HaCaT cell on different concentrations of gelatin on days 1, 4, and 7 (n = 3). (**E**) Proliferation of HaCaT cells on hydrogels with different concentrations of gelatin indicated by relative fluorescence unit using PrestoBlue Cell Viability Reagent (n = 10). Scale bar is 100 µm. (Error bars indicate standard deviation. ns, **, ***, and **** indicate nonsignificant, *p* < 0.01, *p* < 0.001, and *p* < 0.0001, respectively.).

We determined the effects of gelatin concentration on cell viability, attachment, proliferation, and formation of tight junctions using the live/dead assay, cytoskeleton staining, and measuring cell number and coverage area (Figure 4). In three different concentrations of gelatin, over 90% cell viability was achieved (Figure 4A) while maintaining their tightly packed morphology (Figure 4B), exemplifying the biocompatibility of enzymatically crosslinked gelatin. Moreover, measuring the cell covered area with ImageJ software (National Institutes of Health, Bethesda, MD, USA) on day 1 showed 60% surface coverage on 20% gelatin, while in 15% and 10% gelatin the surface coverage was about 40% (Figure 4C), showing better cell attachment in 20% gelatin. This is a result of more available cell binding sites provided by higher gelatin concentrations [23,43].

The numbers of live cells on the hydrogels were counted on days 1, 4, and 7 to evaluate the proliferation per unit area. Results show that the numbers of HaCaT cells on 15% and 20% gelatin on days 4 and 7 were significantly higher than the number cells on 10% gelatin (Figure 4D). This implies that mechanical properties of the substrate significantly affect HaCaT cells proliferation and that cells tend to proliferate more on stiffer surfaces. To confirm this effect, the metabolic activity of HaCaT cells was investigated using PrestoBlue cell viability reagent (Invitrogen by ThermoFisher Scientific, Waltham, MA, USA). After a 45-min incubation of cells with PrestoBlue, relative fluorescence intensity of the supernatants confirmed a higher proliferation rate for 15% and 20% gelatin hydrogels compared to 10% gelatin (Figure 4E). These results accord with previous observations which show the effect of the substrate’s mechanical properties on HaCaT cells proliferation and migration. It has been observed that the rate of cell proliferation and migration was significantly higher on substrates with higher mechanical strength and stiffness [60].

Studying the effects of gelatin concentration on mechanical properties, cell viability, and proliferation reveals that 20% gelatin hydrogel offers higher mechanical strength and support cell attachment and proliferation compared to 10% and 15% gelatins. Therefore, 20% gelatin was selected for developing the epidermal tissue.

### 2.3. Evaluating the Model Function

Following hydrogel characterization and cell compatibility analysis, a functional multilayer epidermis model was developed using 20% gelatin with 5 U/mL TG to provide a higher mechanical stability for long-term differentiation of HaCaT cells. The multilayer construct was formed by culturing HaCaT cells at the air-liquid interface and characterized.

#### 2.3.1. Gelatin Hydrogel Permeability

**Figure 5:**
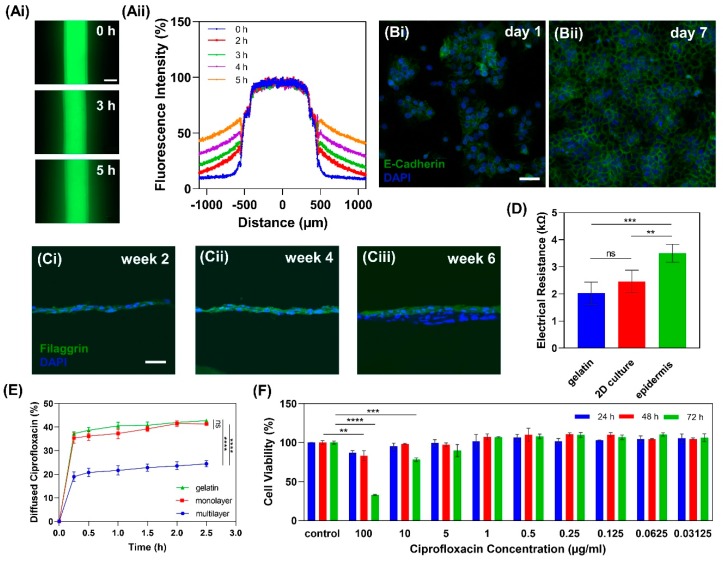
In vitro epidermis model characterization. (**A**) Fluorescein isothiocyanate–dextran (FITC-dextran, 70 kD) diffusion through the channel to 20% gelatin, scale bar is 500 µm. (**B**) Fluorescence image of immunocytochemical staining of *E-cadherin* (green) in HaCaT cell junctions and DAPI for nucleic staining (blue) on day 1 (**i**) and day 7 (**ii**) when cells reached confluency; scale bar is 50 µm. (**C**) Fluorescence image of immunocytochemical staining of filaggrin protein (green, late differentiation marker of HaCaT) and nuclei with DAPI (blue) in week 2 (**i**), 4 (**ii**), and 6 (**iii**); scale bar is 50 µm. (**D**) Electrical resistance measurements (n = 5). (**E**) Ciprofloxacin diffusion from the surface of 20% gelatin to the channel, indicating the barrier function of epidermis (n = 3). (**F**) The cytotoxic effects of different concentrations of ciprofloxacin on HaCaT cells (n = 3). (Error bars indicate standard deviation. ns, **, ***, and **** indicate nonsignificant, *p* < 0.01, *p* < 0.001, and *p* < 0.0001, respectively.).

In order to investigate the permeability of 20% gelatin for media delivery through the hollow channel, the diffusion rate of fluorescein isothiocyanate–dextran (FITC-dextran) with 70 kD molecular size was studied. The result showed that after 5 h, more than 50% of FITC-dextran diffused to a distance of 300 µm from the channel (Figure 5). Considering the distance between the cells on the gel and channel, which was about 200 µm, the permeability of 20% gelatin is adequate for nutrient delivery to the HaCaT cells from the channel.

#### 2.3.2. Cell Tight Junction Analysis

To evaluate HaCaT cells junctions, a confluent monolayer of cells on 20% gelatin was stained with *E-cadherin* antibody. *E-cadherin* is not only necessary for cell-cell adhesion, but it affects various cellular functions such as cell signaling and cytoskeleton regulation [61,62]. The expression of *E-cadherin* shows that after 7 days of submerged culture, HaCaT cells form a confluent monolayer in which cells have tight junctions (Figure 5B).

#### 2.3.3. Multilayer Epidermis Formation

After a monolayer of HaCaT cells was formed within 5–7 days of submerged culture, the medium was removed from the seeding zone and the differentiation medium was delivered to the cells through the channel, maintaining the cells at the air-liquid interface. HaCaT cells’ differentiation and stratification started under the air-liquid-interface condition and lasted for about 6 weeks. Filaggrin as a late epidermal differential marker has a pivotal role in the skin barrier function [63]. To investigate the protein expression of the reconstructed epidermis on 20% gelatin, filaggrin antibody was used to show the terminally differentiated keratinocytes over 6 weeks of differentiation (Figure 5C). The epidermis model exhibited positive expression of filaggrin. However, its expression structure was slightly disorganized, which was likely due to the immature terminal differentiation of HaCaT cells and the absence of other cell types such as fibroblasts [28].

#### 2.3.4. In Vitro Epidermis Barrier Function

To study the barrier functionality of the in vitro epidermis model, the electrical resistance across the multilayered epidermis model was measured. The results show that the epidermis model provides a higher electrical resistance with 3.5 ± 0.3 kΩ compared to the bare gelatin hydrogel with 2.0 ± 0.4 kΩ electrical resistance and 2D cell culture on gelatin with 2.5 ± 0.4 kΩ (Figure 5D). This is likely due to the functional tight junctions of keratinocytes and the presence of filaggrin protein in the epidermis model. However, the lower degree of stratification in differentiated HaCaT cells and the absence of other skin cell types resulted in a resistance on the lower end of the range for human skin, which has an electrical resistance of 1–10 kΩ [28].

In addition to electrical resistance, the drug permeability of the epidermis model was studied by measuring the percentage of penetrated ciprofloxacin from the epidermal surface to the channel. Results show that the epidermis model (24.4%) significantly hinders the drug permeation into the channel when compared to the cell-free gelatin (42.7%) and 2D cell culture on gelatin (41.3%) (Figure 5E).

#### 2.3.5. Drug Cytotoxicity Test

To study the viability of HaCaT cells treated with varying dosages of a drug, cells were treated with different ciprofloxacin concentrations and the viability of HaCaT cells was studied using PrestoBlue cell viability reagent. Ciprofloxacin with 100 µg/mL concentration significantly decreased cell viability after 48 h treatment compared to the control, while 10 µg/mL treatment affected the cell viability after 72 h of exposure. This result is comparable to the results of previous studies of different concentrations of ciprofloxacin on fibroblast cell viability [64,65]. The lower concentration of ciprofloxacin, on the other hand, do not adversely affect the cell viability (Figure 5F).

### 2.4. Wound Infection Modeling

**Figure 6:**
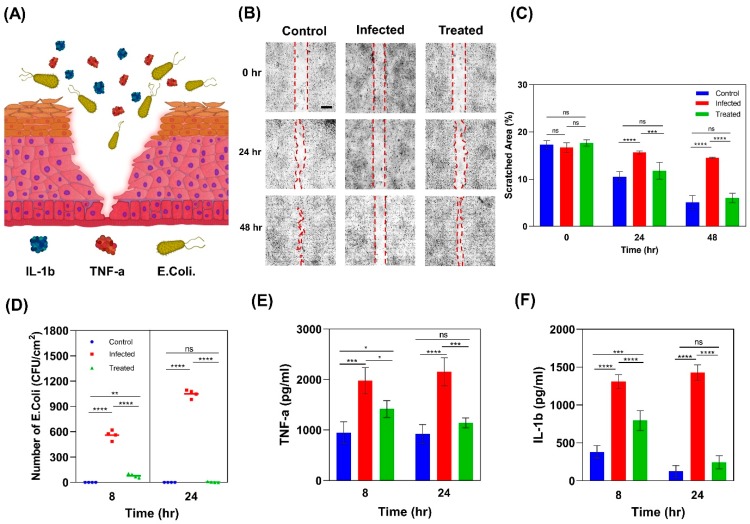
Infected wound model. (**A**) Schematic picture of an infected wound representing keratinocytes’ response to *E. coli*. (**B**) Scratch assay on control, sample infected with *E. coli*, and sample infected with *E. coli* and treated with ciprofloxacin; scale bar is 500 µm. (**C**) The percentage of scratched area using NIH ImageJ software (n = 3). (**D**) *E. coli* colony-forming units (CFU) are numbers per unit cell cultured area at 8 and 24-h (n = 4). (**E**) The expression of TNF-α by keratinocytes in response to infection (n = 3). (**F**) The expression of IL-1β by keratinocytes in response to infection (n = 3). (Error bars indicate standard deviation. ns, *, **, ***, and **** indicate nonsignificant, *p* < 0.1, *p* < 0.01, *p* < 0.001, and *p* < 0.0001, respectively.).

In addition to the physical barrier, the epidermis actively contributes in immunologic barrier function by the expression of pattern recognition receptors (PRRs) on keratinocytes. These receptors recognize pathogen-associated molecular patterns of microorganisms and lead to the expression of pro-inflammatory mediators, such as interleukin 1 beta (IL-1β) and tumor necrosis factor alpha (TNF-α), to activate the early innate immune system (Figure 6A) [50]. Studying the epidermis’s behavior in response to injury and infection can orient researchers to develop more effective therapeutic agents and drug delivery systems.

#### 2.4.1. Scratch Wound Healing Assay

To mimic an infected wound in vitro, a scratch was created on the epidermis construct using a 200 µL micropipette tip, and it was then infected with *E. coli*, one of the most common gram-negative bacteria (Figure 6B). Ciprofloxacin as an antimicrobial agent was used to treat the infected model. Three groups were considered for the wound healing study, including (i) an infected model with 0.4 µg/mL ciprofloxacin treatment, (ii) an infected model without treatment, and (iii) an uninfected model without ciprofloxacin as the control. Scratch closure was monitored using bright field images of the samples taken after 24 and 48 h. Results show that scratches in the control and treated groups closed in a similar timeframe, while the scratch in the untreated infected model remained open (Figure 6C).

#### 2.4.2. Colony Forming Unit Counting

To investigate the effectiveness of ciprofloxacin treatment, swabs were taken from samples after 8 and 24 h and cultured on nutrient agar sheets. After 24 h of incubation of the agar sheets, colony-forming units (CFU) were counted as an indicator of the number of bacteria. Results showed that at 8-h time point, there were ≈600 CFU/cm^2^ in the infected samples (Figure 6D). This number grew to ≈1000 CFU/cm^2^ after 24 h of exposure to *E. coli*. Meanwhile in the control and treated samples, no significant bacteria colonies were observed. This confirmed that ciprofloxacin was effective for treating infections from *E. coli*.

#### 2.4.3. Pro-Inflammatory Response

The inflammatory response serves as protection to restore the homeostatic state after a harmful disturbance. Infections can activate the innate immune system to defend against the invading pathogen by stimulating the expression of pro-inflammatory cytokines such as IL-1β and TNF-α by keratinocytes [66]. IL-1β and TNF-α function through autocrine signaling on keratinocytes and result in the expression of other pro-inflammatory cytokines, such as IL-6 and IL-8 [67]. To investigate the pro-inflammatory response of keratinocytes to infection, we analyzed the TNF-α and IL-1β expression in HaCaT cells infected with *E. coli*. The results show that after 8 h exposure to *E. coli*, HaCaT cells in infected samples expressed significantly higher levels of IL-1β (1311 ± 90 pg/mL) and TNF-α (1977 ± 262 pg/mL) with respect to the control group (383 ± 80 pg/mL IL-1β and 949 ± 215 pg/mL TNF-α). These levels further increased after 24 h of exposure to *E. coli* (Figure 6E,F). In accordance with the present results, previous studies have demonstrated that after a few hours of bacteria induction, HaCaT cells started producing TNF-α and IL-1β in response to infection and the level of both cytokines increased over 24 h [68,69,70,71]. Treatment with 4 µg/mL ciprofloxacin reduced the expression levels of IL-1β and TNF-α in the infected samples to 795 and 1417 pg/mL, respectively. However, the expressed levels of IL-1β and TNF-α were still significantly higher than the control group. After 24 h of treatment, however, the levels of TNF-α and IL-1β decreased to 246 and 1142 pg/mL, respectively, and settled at the same levels as for the control group. The trend of pro-inflammatory cytokines’ expression is consistent with CFU results and confirms the effectiveness of ciprofloxacin in treating *E. coli* infection and terminating the inflammatory response of keratinocytes.

## 3. Experimental Section

### 3.1. Preparation of Gelatin Hydrogel

Enzymatically crosslinked gelatin was prepared as described previously [45]. Briefly, gelatin powder from porcine skin (Sigma-Alrich, St. Louis, MO, USA) was dissolved in Dulbecco’s phosphate-buffered saline (DPBS, Sigma-Aldrich) at 60 °C to achieve final gelatin concentrations of 10%, 15%, and 20% (w/w). Then the solutions were sterilized using 0.22 µm filters. The transglutaminase (TG, Modernist Pantry, Eliot, ME, USA) solutions were prepared with different concentrations of 2.5, 5, and 10 U/mL by dissolving proper amount of TG in PBS and then sterilized using 0.22 µm filter. Gelatin/TG hydrogels were prepared by mixing TG solutions with different concentrations of gelatin at 60 °C. Then solutions were incubated at 37 °C for 12 h crosslinking process.

### 3.2. Mechanical Properties Measurement

For optimizing the amount of enzyme, the storage modulus of 15% (w/w) gelatin with various amounts of TG was measured using the non-destructive method described previously [58]. Briefly, 2 mL of 15% gelatin solutions with 2.5, 5, and 10 U/mL TG were poured in the detachable sample holder specially designed for ElsatoSens™ Bio2 (Rheolution, Montreal, Quebec, Canada), and real-time storage modulus measurements were performed for 12 h using ElastoSens™ Bio2. Using the device, for each sample, the storage modulus was measured 3 times every 5 min. Samples were prepared in 3 replicates for each condition.

### 3.3. Swelling Ratio

Gelatin hydrogel disks were freeze-dried, weighed (*W_d_*), and incubated in DPBS at 37 °C for 24 h. At each time point, they were removed from DPBS, lightly blotted, and weighed (*W_s_*). The swelling ratio of the swollen gel was calculated according to Equation (1) [28]:

(1) $$\mathrm{Swelling}\;\mathrm{Ratio}\;(\%)=\frac{W_{s}-W_{d}}{W_{d}}\times 100$$

### 3.4. In Vitro Enzymatic Degradation

For studying the degradation rates of gelatin hydrogels, gelatin disks with 1.5 cm diameter were prepared as mentioned above. After gelation, the hydrogels were weighed (*W*_0_) and immersed in 2 mL of 2 U/mL collagenase (Sigma-Aldrich) solution in 12-well plates and incubated at 37 °C. Weight measurements (*W_t_*) were performed every 24 h for 20 days. The collagenase solutions were refreshed every 2 days. Finally, the degree of degradation was plotted as the percentage of the remaining hydrogel mass versus the initial hydrogel mass according to Equation (2) [28]:

(2) $$\mathrm{Mass}\;\mathrm{Remaining}\;(\%)=\frac{W_{0}-W_{t}}{W_{0}}\times 100$$

### 3.5. Mechanical Stability of Gelatin Hydrogel in Culture

In order to examine the mechanical behaviors of gelatin in culture, immortalized human keratinocytes (HaCaTs, Addexbio, San Diego, CA, USA) were seeded with the seeding density of 50,000 cells/cm^2^ onto the hydrogels prepared in the detachable sample holder. Subsequently, the samples were incubated at 37 °C and 7% CO_2_ for cell attachment for 24 h. The ElastoSens™ device was used for measuring the storage modulus of hydrogels over 14 days of culture. The measurement was carried out for three replicates at each condition.

### 3.6. Scanning Electron Microscopy

Scanning electron microscopy (SEM) was used to visualize the morphology of gelatin. For this purpose, the lyophilized hydrogels were mounted on the SEM stub and coated with gold-palladium hummer sputter (Hummer VI sputter coater, Anatech USA, Hayward, CA, USA). SEM images were obtained by a Hitachi electron microscope (Hitachi S4800, Tokyo, Japan) with 1.0 kV voltage.

### 3.7. Cell Attachment and Cell Number

Gelatin/TG solutions with various gelatin concentrations (10, 15 and 20%) were prepared as described above. After incubating the hydrogels with Dulbecco’s modified eagle’s medium (DMEM, Gibco^™^ by ThermoFisher Scientific, Waltham, MA, USA) with 10% fetal bovine serum (Gibco™ by ThermoFisher Scientific, Waltham, MA, USA) for 12 h, HaCaTs were seeded onto the gelatin hydrogels with the seeding density of 50,000 cell/cm^2^. After 24 h, cells were stained with live/dead viability kit (Invitrogen by ThermoFisher Scientific, Waltham, MA, USA) to assess cell adhesion by measuring the percentage of cell-covered area. In order to stain the samples, hydrogels were washed three times with sterile PBS and incubated at room temperature for 30 min in a solution with the concentration of 2 μM calcein— acetoxymethyl (calcein—AM) and 4 μM ethidium homodimer in DPBS. After incubation, the samples were washed with PBS and then images were obtained by ZEISS confocal microscope (Zeiss LSM880, Carl Zeiss Microscopy, Jena, Germany). Additionally, ImageJ software (National Institutes of Health, Bethesda, MD, USA) was used for determining the cell-covered area. Similar staining procedure was utilized on days 1, 4, and 7 to determine the cell number. Samples were prepared in 3 replicates for each gelatin concentration.

### 3.8. Cell Morphology

To study the morphology of HaCaT cells on gelatin hydrogels, the samples were prepared as described previously, and after 24 h incubation, cells were fixed with 3.7% (v/v) formaldehyde (VWR, Radnor, PA, USA) for 15 min. Then, cells were washed with PBS and permeabilized with 0.1% (v/v) Triton X-100 (BIO BASIC, Markham, Ontario, Canada) solution for 15 min. Afterward, the samples were washed with PBS and incubated with 0.5% 4′,6-diamidino-2-phenylindole (DAPI, Sigma-Aldrich, St. Louis, MO, USA) and 0.1% Alexa Fluor™ 488 Phalloidin (Invitrogen by ThermoFisher Scientific, Waltham, MA, USA) solution in PBS for 30 min. Finally, the samples were washed and imaged using a Zeiss confocal microscope (Carl Zeiss Microscopy, Jena, Germany) with 20× magnification objectives.

### 3.9. Cell Proliferation

In order to evaluate cell proliferation on different concentrations of gelatin, the samples were incubated with media containing PrestoBlue reagent (Invitrogen by ThermoFisher Scientific, Waltham, MA, USA) with 9:1 ratio for 45 min at 37 °C on days 1, 4, and 7. Afterwards, 100 µL of supernatant was collected from each well and the fluorescence intensity was measured at excitation wavelengths of 560 nm and emission of 590 nm using a microplate reader (Infinite M Nano, Tecan, Tecan Trading AG, Zurich, Switzerland). Relative proliferation rate was calculated by normalizing the measured intensity at each condition with respect to the condition with the lowest intensity.

### 3.10. Model Development

To fabricate the vasculature-embedded hydrogel, a layer of 20% gelatin was loaded into a 35 mm petri dish. After 30 min of incubation at room temperature, 38% Pluronic F127 (Sigma-Aldrich) solution was printed onto the gel as a sacrificial material using a 3D printer (CELLINK+, CELLINK, Gothenburg, Sweden) with 280 kPa pressure. Immediately after printing the pattern, another layer of gelatin was poured onto the construct and the hydrogel was incubated at 37 °C for 12 h.

### 3.11. Gelatin Hydrogel Permeability

To show the permeability of gelatin hydrogel in nutrient delivery from the channel to the HaCaT cells, the diffusion rate of 70 kD FITC-dextran (Sigma-Aldrich) was measured by live imaging. For this purpose, 2 mg/mL solution of FITC-dextran was loaded into the model’s channel under an Axio Observer ZEISS microscope (Carl Zeiss Microscopy, Jena, Germany). Images were taken at 15 min intervals for 5 h.

### 3.12. Cell Tight Junction Analysis

To characterize the formation of tight junctions after 7 days of submerged culture, the cells were immunostained with *E-cadherin* and imaged. Briefly, cells were fixed with 3.7% formaldehyde solution for 15 min; the samples were incubated with blocking buffer (5% bovine serum albumin and 0.3% Triton X-100 in PBS) for 60 min at room temperature. After washing the samples with PBS, they were incubated with *E-cadherin* antibody dilution buffer (Rabbit mAb Alexa Fluor^®^ 488 Conjugate, Cell Signaling Technology, Danvers, MA, USA) at 4 °C overnight. Subsequently, the wells were incubated with DAPI solution (5 µg/mL) for 15 min at room temperature. The cells were then washed with PBS and imaged using confocal microscopy.

### 3.13. In Vitro Epidermis Model Development

HaCaT cells were seeded onto the seeding zone with density of 50,000 cells/cm^2^. After 7 days, when the cells reached confluency, the differentiation process was induced as described previously [72]. Briefly, the medium was removed, and the cells were left at the air-liquid-interface. The differentiation serum-free medium composed of DMEM, 1.8 mM Ca^2+^ (Sigma-Aldrich), 2 ng/mL TGF-α (Sigma-Aldrich), and 100 ng/mL GMCSF (R&D Systems, Minneapolis, MN, USA) was delivered to the cells through the channel.

### 3.14. Protein Expression of Developed Epidermis Model

Differentiation of HaCaT cells was studied by immunostaining of the epidermal cross-sections with filaggrin antibody (terminally differentiation marker) and imaged. To prepare the cross-sections, the epidermis model was fixed with 3.7% formaldehyde solution for 15 min, and then left in a 30% sucrose solution over night at 4° C. Then, the hydrogel was mounted in an optimal cutting temperature (OCT) compound and frozen using liquid nitrogen prior to cryostat sectioning. Sections with 5 µm thickness were collected on positively charged glass slides. Then, the sections were permeabilized using 0.5% Triton X-100 in PBS solution for 5 min at room temperature. After 3 rounds of washing with PBS, samples were blocked using 5% normal goat serum albumin solution for 1 h at room temperature. Then, they were incubated with filaggrin antibody dilution buffer (Mouse mAb Alexa Fluor^®^ 488 Conjugate, Novus Biologicals, Littleton, CO, USA) at 4 °C overnight. Subsequently, the samples were incubated with DAPI solution (5 µg/mL) for 15 min at room temperature. The samples were then washed with PBS and imaged using confocal microscopy.

### 3.15. In Vitro Epidermis Electrical Resistance

For measuring the electrical resistance of the developed epidermis tissue, samples were prepared as described before. After 6 weeks of culturing, the cell-seeded area was cut with the dimensions of 1 cm × 1 cm. The electrical resistance of the epidermis model was directly measured using a multimeter (Fluke 87). The controls were 20% cell-free gelatin and gelatin with a confluent cell monolayer. Samples were prepared in 5 replicates.

### 3.16. In Vitro Epidermis Drug Permeability

To examine the permeability of epidermis model, 500 μL of 1 µg/mL ciprofloxacin (Sigma-Aldrich, USA) in cell culture media was added onto the seeding zone above the epidermis layer, and then the model was incubated at 37 °C. At different time points (0.25, 0.5, 1, 2, and 2.5 h), the medium from the channel was transferred into a 96-well plate, and the channel was filled with fresh media. Quantity of diffused ciprofloxacin into the channel was measured using a microplate reader at excitation wavelength of 270 nm and emission wavelength of 445 nm. Ciprofloxacin standard curve was plotted as fluorescence intensity versus different ciprofloxacin concentrations at 0.03125, 0.0625, 0.125, 0.25, and 0.5 µg/mL (data not shown). Equation (3) was used to determine the percentage of diffused ciprofloxacin in the samples [31].

(3) $$\mathrm{Diffused}\;\mathrm{ciprofloxacin}\;(\%)=\frac{C_{t}V_{t}}{C_{0}V_{0}}\times 100$$

where *C_t_* is the ciprofloxacin concentration of samples calculated by a comparison with the standard curves and *V_t_* is the volume of solution removed from the channel. *V*_0_ is the total volume of ciprofloxacin solution (500 µL) with the concentration of *C*_0_ in the seeding zone (1 µg/mL).

### 3.17. Drug Cytotoxicity Test

To determine the cytotoxicity effects of different concentrations of ciprofloxacin, the HaCaT cells were seeded with a cell density of 50,000 cells/cm^2^. Ciprofloxacin solutions in cell culture media were prepared with concentrations of 100, 10, 5, 1, 0.5, 0.25, 0.125, 0.0625, and 0.03125. After differentiation, the cells were treated with ciprofloxacin solutions for 3 days. The viability of cells was investigated after 24, 48, and 72 h post treatment using PrestoBlue Cell Viability Reagent. Relative viability was calculated by normalizing the fluorescence intensity of each condition with the control condition (no drug).

### 3.18. Bacterial Study

HaCaT-seeded gelatins were prepared, and then the media were removed and substituted with 1 mL of fresh antibiotic-free media two days before infecting with bacteria. A single colony of *Escherichia coli* (*E. coli*, W3110, ATCC, Manassas, VA, USA) was inoculated into 10 mL LB broth (Difco™ LB Broth, Lennox, Thermo Fisher Scientific, Waltham, MA, USA) culture medium overnight. After being incubated and reaching OD600 of ≈0.4, a bacterial solution with the concentration of 10^8^ cells/mL was prepared. Three groups of samples were prepared: (i) a control group treated with 25 µl/sample bacteria-free PBS; (ii) (iii) treated groups incubated with 25 µL of bacteria solution. After 2 h of incubation, the media of each group were substituted with the fresh media to remove the non-adherent bacteria, and 10 µL of ciprofloxacin solution with the concentration of 400 µg/mL was added to the third group.

### 3.19. Scratch Wound Healing Assay

To study the healing of infected and treated wound models, samples were prepared as described above and a scratch was created on the epidermis construct using a 200 µL micropipette tip and then infected with *E. coli*. For the purpose of studying the efficacy of treatments on cell migration, samples were imaged at 0, 24, and 48 h. Images were taken by microscope using bright field and cell migration activity was evaluated by measuring the scratched area using Image J software. For each well, three images were taken from randomly selected areas. Experiments were conducted independently in triplicate.

### 3.20. Pro-Inflammatory Cytokine Analysis

To study the pro-inflammatory response of the epidermis model to infection, samples were prepared as described above in two sets, one for collecting the supernatant and the second for culturing the swabs from each sample on nutrient agar sheets at 8 and 24 h for CFU counting. The expressions of two pro-inflammatory cytokines, IL-1β and TNF-α, were measured using Human Mini ABTS ELISA Development Kit (Pepro Tech, Rocky Hill, NJ, USA).

### 3.21. Statistical Analysis

Results were analyzed by GraphPad Prism version 8 (GraphPad Software, San Diego, CA, USA). Statistical significance was analyzed using one-way analysis of variance (ANOVA) for more-than-two-group comparisons with one independent variable and two-way ANOVA for more-than-two-group comparisons with two independent variables.

## 4. Conclusions

In this study, we developed a simplified, functional in vitro infected wound model for drug screening. The epidermis construct was used for infected wound modeling and studying the skin’s pro-inflammatory response. The model consisted of a keratinocyte layer formed on a gelatin matrix. The effects of gelatin and transglutaminase concentrations were studied in terms of the mechanical properties of gelatin hydrogel and the subsequent cell attachment and proliferation. Results showed that the 20% gelatin hydrogel strongly supported cell attachment and proliferation and showed significantly prolonged degradation compared to the lower concentrations. Therefore, the higher concentration of gelatin (20% w/w) was selected for the model formation. HaCaT cells were cultured on the model for 7 days submerged in culture media, followed by an additional 6 weeks of culture at the air-liquid interface. The results showed that HaCaT cells terminally differentiated and formed the multilayer structure of the epidermis. The epidermis model showed the barrier functions in terms of electrical resistance and drug permeability. Although this epidermis model lacked complete stratification and cell organization due to the absence of fibroblast cells in the construct, it is still a functional in vitro construct for wound modeling and drug testing. The in vitro epidermis model was used for studying the pro-inflammatory response of HaCaT cells to infection, and the effectiveness of ciprofloxacin in infection treatment. The developed epidermis model has great potential for large-scale infected wound modeling, studying drug cytotoxicity, and developing trans-epidermal drug delivery systems because of its functionality, low fabrication cost, and reproducibility.
